# The Immune System, Immunity and Immune Logics: Troubling Fixed Boundaries and (Re)conceptualizing Relations

**DOI:** 10.17157/mat.11.1.9443

**Published:** 2024-03-15

**Authors:** Andrea Ford, Julia Swallow

How did we come to believe that as living beings, ‘the body’ separates us from each other and from the world rather than connects us?

—Ed Cohen, *A Body Worth Defending.*

## Introduction

Immunity, though a metaphor, is naturalised in contemporary biomedicine: ‘immunology’ and the ‘immune system’ are taken-for-granted domains that have seen remarkable amounts of investment in recent years. This has led to developments in understanding, managing and treating a wide variety of conditions, from HIV/AIDS to rheumatoid arthritis. Immunologist Daniel Davis for instance describes the immune system as the ‘key to human health,’ and ‘a revolutionary new approach to medicine and well-being’ (Davis 2018). This renewed biomedical attention has been brought sharply into public discourse during the ongoing COVID-19 pandemic, with gestures beyond humans towards zoonotic disease and a ‘One Health’ paradigm. And yet immunity is often (biomedically) framed as a project of individual self-investment: what Cohen (2009) calls ‘biopolitical individualisation’ (cf. Martin 1994; Rose 2007; Brown 2019). This line of thinking about immunity focuses on shoring up the boundaries, defences, and capacities of individual bodies, whether by means of pharmaceutical or other technologies, or practices of self-care and self-maintenance.

Whatever way it’s invoked, immunity is always a question of relations. As Ed Cohen elaborates in his ‘immunophilosophical’ genealogy, *A Body Worth Defending,* ‘immunity’ is the etymological inverse of ‘community’ (2009, 29). Until about 150 years ago, the notion of immunity referred to legal protection from political and economic damage, to entitlements that exempted people or collectives from legal obligations that would otherwise be universal. Biological immunity to disease within individual bodies has taken up this political legacy, and become the dominant understanding of the term, while bearing within itself a profoundly ideological heritage (Cohen 2009; Biss 2015). Most broadly, immunity is about the possibility of protection; about whether and how dangers and harms can be prevented. The desire to protect by drawing and defending boundaries seems commonsensical—it is taken for granted in many Euro-American contexts (Ford, forthcoming). But defending boundaries is not necessarily possible or desirable; it is neither a fully accurate metaphor for existing in the world nor a viable strategy for addressing imminent political, ecological, and medical challenges.

The argument of this special issue is that empirical contexts where ‘immunity’ is contested are attempts to manage the fact that we are constantly *in relation* to other people and other beings. Contemporary instantiations of immunity grapple with the fact that we are part of our context and not separate from it. This manifests in biomedical discoveries and paradigm shifts: Davis documents how new versions of immunology account for a system ‘in constant flux’ depending on the time of day, weather, stress, or age, and that ‘layer upon layer of biological checks and balances’ regulate how the immune system negotiates and targets ‘what’s not part of you’ (2018, 3). He suggests that the immune system ‘is more pluralistic than we currently imagine’, calling it ‘enigmatic’ and full of potential for designing new tailored medicines. Yet the problem of context also manifests through people pushing against biomedical paradigms of immunity. If, as Cohen (2009, 8) argues, ‘bioscience affirms that living entails a ceaseless problem of boundary maintenance’, then numerous instances of boundary crossing and enmeshed ecologies destabilise this view of life and living.

To make this argument, the special issue brings together scholars from anthropology, sociology, science and technology studies (STS) and geography who are conducting empirical work on immunity, the immune system, and immune logics in contemporary settings. The collection builds upon an interdisciplinary workshop held in February 2021 that drew contributors from Finland, France, Germany, Japan, Norway, the UK and the US, and makes a case for the timeliness and broad relevance of immunity in its evolving manifestations. In the face of ongoing pandemics, climate devastation, compounding forms of toxicity, antimicrobial resistance, and other crises that force us to confront our inseparability from what surrounds us, immunity is not so much changing from one thing into another, but a concept that encapsulates a wide variety of contemporary tensions which sit at the intersections of the biological, social and political realms.

## Immunity and metaphors

Social science analyses of immunity have long brought medical approaches to immunity into conversation with the ways immunity circulates as a logic present outside medical settings (Haraway 1992; Martin 1994, 1990; Napier 2002, 2012; Biss 2015; Brown 2019). As noted above, the concept of immunity is more deeply political than it is medical, and when applied in biological contexts is, itself, a metaphor. Numerous subsidiary metaphors are key to the bridges social scientists have analysed in immunology, from ‘defence’ to ‘flexibility.’ Such analyses emphasise the mutual shaping of science with, and as, culture (Weiss 1997; Henry 1999; Martin 1998; Martin 2010; Swallow 2023) and highlight how metaphors are politically charged (see Larson, Nerlich and Wallis 2005; Wallis and Nerlich 2005).

Militaristic metaphors dominate biomedical discourse on the immune system, emphasising fixed dichotomies such as self/other and internal/external (e.g., Cohen 2009; Haraway 1991; Martin 1990, 1994; Napier 2002). The body is represented as an ‘embattled self’ (Jamieson and Blackman 2015, 108) with scholars examining how ‘self’, the body, and identity are entwined in empirical sites ranging from organ transplantation (Martin 1994) to antimicrobial resistance (Cohen 2009; Davis et al. 2016; Martin 1994; Napier 2002). Alongside these militaristic metaphors the agility, adaptability and resilience of the immune system is also foregrounded (see Napier 2002). And yet the metaphor of defence and boundary maintenance has been undermined by recent immune science that allows for more complexity in how self versus non-self is determined within the body.

Anthropologists have interpreted this shift as challenging Enlightenment legacies of how ‘self’, ‘identity’, and ‘foreignness’ are construed (Napier 2012, 123). Indeed, as a cultural problem and value, ‘immunity’ reflects a troubling reliance on the concept of an autonomous individual, which is foundational to both Enlightenment concepts like personal rights and new neoliberal imperatives like ‘responsibilization’ (Rose 2007). The individual is the supposedly coherent unit whose boundaries can be defended, a form on an environmental field. Yet as Ford describes, contemporary transgressive figures such as microbes, chemicals, and stress blur the separation between a human and their environment, enacting ‘embodied ecologies’ (2019a, 2019b). Humans are part of networks of connection and dependency, and our bodies themselves are environments for other beings within these networks. If one is ‘in’ an environment, then harmful aspects of that environment can be blocked—destroyed, even. Immunity is possible. And likewise, beneficial aspects of that environment can be harnessed, hoarded, commodified (‘nature’ can only be a resource *for* humans insofar as it is understood as not human). But if one is part of an ecology, the environment is not ‘out there’. Rather, it *is* us. We are inextricable from what surrounds and composes us. We don’t exist without it.

Feminist STS scholars have also troubled self/non-self distinctions, and the primacy of the individual, suggesting that these do not align with ‘biological thinking about how organisms coexist in shared ecologies, sometimes with great mutual benefit, sometimes pacifically, sometimes indifferently, and sometimes deleteriously’ (Cohen 2009, 8). Aryn Martin’s (2010) work on foetal-maternal microchimerism is significant here (see also Colls and Fannin 2013; Matzinger 2002). Martin argues that the cell trafficking between the foetus and pregnant body troubles the self/other distinction that permeates immunology discourse, and calls instead for the need to pay attention to productive relations of self and other through attending to relationality (2010). More recently, in the context of antimicrobial resistance, Davis et al. (2016) suggest that ‘this erosion of the absolute in [self/non-self] implies . . . “immune-cosmopolitanism,” that is, an understanding of one’s immunity as reliant on productive relations and amicable coexistence with the other’ (2016, 135; see also Shildrick 2014). Building on this work, in the context of cancer and immunotherapy treatments, Swallow (2023) extends feminist analyses of immunology discourse. By addressing the material stakes of discursive shifts, she calls for attention to patients’ day-to-day experiences of treatment. The discursive framing of immunotherapy brings into being new forms of embodied patienthood in the context of cancer, revealing the exclusions and tensions at work in the entanglements of relationality (see also Giraud 2019).

## Immunity and Relations

Immunity is a way of approaching relations. As conceptual and theoretical tools, both ‘relations’ and ‘immunity’ are continually evolving. ‘Relations’ have long been at the forefront of anthropological perspectives on medicine, and this collection builds upon this legacy. In many ways, immunity is the paradigmatic case for navigating the relationship between individual protections and social obligations, boundaries between self and other, and bodily coherence versus relational entanglement. Anthropological scholarship, including feminist and anti-colonial STS analyses, has continually highlighted the many manifestations of relational entanglement (see Strathern 2020), troubling the intellectual legacies which foreground individuals and that are deeply rooted in contemporary biomedicine (e.g., Weasel 2001, Marsland 2012; Napier 2012; Yates-Doerr 2012; Heinemann 2013; Hicks 2014). The papers in this Special Issue further contribute to this body of work and explore how conflicting conceptual paradigms are being navigated in practice. They raise (and answer) questions about how we conceptualise and act upon risk, health, illness, disease, bodies, and embodied experiences in the contemporary moment.

Among the contributions, concern with relations appears in diverse discussions of social responsibility for health, but equally in the interdependence of bodily systems. Spatial relations consider the often toxic environments in which our bodies are enmeshed (Murphy 2006; Shapiro 2015; Hoover 2017; Ford 2019a; Lock 2019). Temporal relations are present in nostalgic ideas of ‘the natural’, or in the way memory is embodied or toxicity is latent; they show up in discourses about progress and evolution, as well as transgenerational disease transmission (including via epigenetics; see Lock 2015; Lamoreaux 2016) and the chronicity of many contemporary disease conditions. Increasingly immunity is framed as processual and ongoing, versus a fixed and achievable state, which draws our attention to how relations shift and adapt over time.

Emphasising relationality and context pushes against a biopolitical discourse of immunity that promotes thinking about individual prerogatives instead of social solidarity. However, just as there are limits to assessing situations in terms of individuals, there are limits within relational approaches—what might be excluded or foreclosed by foregrounding some relations over others (see Giraud 2019)? Relations are inflected through the politics of race, gender, age, and class (see Williams 2018; Yates-Doerr 2019). New immunitary technologies reassert, but also reconstitute, how we understand race (Wade 2014). Relations are also affective (Ahmed 2004); fear of external threats can be more palatable than embracing the ambiguities inside of us, an insight as applicable to international politics as to intimate exposures. Relations with non-humans are garnering ever more attention in the era of the microbiome and zoonotic diseases, challenging entrenched ideas about environmental domination (Lorimer 2018; Fearnley 2020); yet this can detract attention from vast disparities in human need in a still-colonial world (Wynter 2003; Lewis 2017). The pieces collected here highlight the stakes of ways people are playing with and pushing against the conventional boundaries of individual bodies.

For example, Greenhough and her colleagues Lorimer, Jokela-Pansini, and Kirksey share ‘field notes from a dirty parenting project’ in their Field Note ‘Mapping Microbial Selves’. It considers growing interest in the microbiome as a source of ‘wild immunology’ amidst heightened sensitivity to microbial threats, in the wake of the ongoing COVID-19 pandemic and widespread concerns about antimicrobial resistance. Yet in contrast to work which emphasises the role of expert knowledges and scientific research in shaping microbial norms—distinguishing the ‘good’ germs from the ‘bad’—this Field Note and its accompanying images explore ‘body mapping’ as a way to engage with families to explore their collective understandings of their children’s microbiomes. The body mapping method sensitises the researchers to the familial co-constitution of microbiomes and microbial knowledges, and the striking visual lexicon people draw on to articulate their understanding of their microbiome, pluralising and democratising immunological knowledge.

In another exposition of a multimodal epistemology, Filipe’s Research Article ‘Life-lines’ describes COVID-19 outbreaks and ecologies of support amidst global travel as shared immunities. She uses the metaphor of ‘living on the line’ for the ways in which we enacted, observed, moved, and lived along an unexpected set of lines during the height of the COVID-19 pandemic: from epidemiological charts and curves and the stripes of positive LFTs, to border restrictions and quarantine/isolation mandates, to networks of communication and support that marked everyday living throughout this period. Such material-semiotic lines reflect what Roberto Esposito (2011) calls the logic of immunity: that which protects an organism-system from danger through exceptions and demarcations between Self and Other. Yet Filipe explores how such lines can (and must) be broken and transgressed, combining personal reflection with notes, vignettes, and portraiture in a mode she calls ‘multimodal autoethnography’.

Explicitly tackling ‘Relations as Immunity’, Kenney and Müller write ‘toward community resilience’. Their piece reflects on the charisma and political lability of ‘resilience’ in the early 21st century, a term that originated in the disciplinary context of ecology and now commonly refers to the ability to thrive in the face of trauma and adversity. They embrace and unpack the ambivalence of resilience: on the one hand, resilience can easily be enrolled in neoliberal discourses that demand that individuals protect themselves in the absence of state or community support, while on the other, it can be an important corrective to narratives that focus on damage and attend instead to our innate ability to heal. They illustrate this through fieldwork in the US Pacific Northwest with actors in education and juvenile justice addressing adverse childhood experiences (ACEs).

Taking biosocial relations into the realm of disease, in her Field Note Ford reflects on endometriosis as an inflammatory condition encompassing social, biological, and ecological influences. Endometriosis is a chronic pain condition where tissue similar to the uterine lining develops outside the uterus; although common, its aetiology is poorly understood, and diagnostics and treatments are highly inadequate. Inflammatory response plays a key role in current efforts to recategorise and reconceptualise the disease. Ford considers disease categorisation through her fieldwork with patients and clinicians in and around Edinburgh, Scotland, interrogating how the lived experience of endometriosis challenges ingrained ways of thinking about the body and bodily ‘systems,’ which are reflected in the design of healthcare systems. Endometriosis challenges common metaphors used to describe immune response, as it cannot be described as self-versus-non-self, nor even self-attacking-self (as in auto-immune conditions) but invites something like a ‘self-out-of-place’.

Buer’s Research Article ‘Bio-imaginaries’ approaches immune disease from the perspective of antibody-based pharmaceuticals, which likewise focuses on indeterminate aspects of biomedicine. These monoclonal antibody pharmaceuticals, used to treat rheumatoid arthritis and a number of other conditions, are made from combinations of mouse and human DNA. Buer’s piece shows how the common practice of calling such pharmaceuticals ‘biologics’ is far from a question of mere semantics. Through following a pregnant person who has been hospitalised on a Norwegian rheumatology ward and is weighing the debilitating consequences of her disease against concerns about pharmaceutical risks for herself and her unborn child, Buer elaborates her epistemological creativity as part of a complex project of semantics where analogies and oppositions of biologic and chemical, natural and man-made, health and unhealth work to render some knowledge plausible and some implausible. Such ‘semantic economies’ illustrate how pharmaceuticals are produced as safe and efficacious alongside other meanings and values.

Keeping on the topic of pharmaceuticals, Oikkonen writes about vaccines, the quintessential vehicle for immune logics, and how growing concerns over vaccine hesitancy and refusal across the global north have made the introduction of novel vaccines, such as that for COVID-19, a precarious task. Her piece explores the routinisation and rejection of new COVID-19 vaccines through an analysis of public debates in Finland, in a context of international news media and announcements by the European Medicines Agency (EMA) and the World Health Organization (WHO), about three different COVID-19 vaccines, delving into the challenge of convincing the public that a new vaccine is needed, that it is efficient, and that it is safe. Finnish materials are analysed in relation to international news media and announcements by EMA and the WHO, illuminating how international debates about new vaccines are taken up and translated into national public health planning. These processes reshape ideas of immunity, risk and contagion, influenced by affects such as doubt, anxiety and hope that require a cultural analysis of each vaccine as an affective, technoscientific object.

As a whole, the collection gestures towards the multiplicity and complexity of immunity whilst foregrounding the importance of relations in contemporary biomedical issues. This ‘immunological relations’ lens is key to revealing the politics inherent in biomedicine as it becomes ever more central to human lives. This lens allows for a deeper interrogation of the coproduction of immunological knowledge within and outside scientific spaces, which is key to democratising knowledge, and for showing how networks and forms of community exclude and foreclose as much as they invite new forms of (bio)solidarity. The breakdown of binary distinctions, which manifests in indeterminacies in existing forms of immunological knowledge, also brings into being (new) disease ontologies which reshape how disease is experienced and managed. Foregrounding immunological relations also draws attention to the wider political economy of biomedicine, including normative assumptions around vaccine development and uptake. Emphasising immunity as relations across these pieces illuminates the dynamism that is necessary to navigate shifting cultural frames. Immunity therefore remains important to interrogate as both an empirical site and conceptual tool. At the centre of this epistemological and ontological work is the need to trouble individualism, which itself is a political task with the potential to exclude and foreclose as much as it generates.
